# Serum amyloid A and Janus kinase 2 in a mouse model of diabetic kidney disease

**DOI:** 10.1371/journal.pone.0211555

**Published:** 2019-02-14

**Authors:** Brad P. Dieter, Rick L. Meek, Robert J. Anderberg, Sheryl K. Cooney, Jen L. Bergin, Hongyu Zhang, Viji Nair, Matthias Kretzler, Frank C. Brosius, Katherine R. Tuttle

**Affiliations:** 1 Providence Medical Research Center, Providence Health Care, Spokane, Washington, United States of America; 2 Internal Medicine, University of Michigan, Ann Arbor, Michigan, United States of America; 3 Molecular and Integrative Physiology, University of Michigan, Ann Arbor, Michigan, United States of America; 4 Institute of Translational Health Sciences, Kidney Research Institute, Nephrology Division University of Washington, Seattle, Washington, United States of America; University of Houston, UNITED STATES

## Abstract

**Background:**

Serum amyloid A (SAA), a potent inflammatory mediator, and Janus kinase 2 (JAK2), an intracellular signaling kinase, are increased by diabetes. The aims were to elucidate: 1) a JAK2-mediated pathway for increased SAA in the kidneys of diabetic mice; 2) a JAK2-SAA pathway for inflammation in podocytes.

**Methods:**

Akita diabetic mice (129S6) with podocyte JAK2 overexpression and angiotensin II infusion (4 weeks) were given a JAK1,2 inhibitor (LY03103801, 3 mg/kg/day orally for the last two weeks). Kidneys were immunostained for SAA isoform 3 (SAA3). SAA3 knockout and control mouse podocytes were exposed to advanced glycation end products (AGE) or exogenous SAA with JAK2 inhibition (Tyrphostin AG 490, 50μM). JAK2 activity (phosphorylation, Western blot, 1 hour) and mRNA for SAA3 and associated inflammatory genes (Cxcl5, Ccl2, and Ccl5) were measured by RT-PCR (20 hours).

**Results:**

SAA3 protein was present throughout the diabetic kidney, and podocyte JAK2 overexpression increased tubulointerstitial SAA3 compared to wild type diabetic controls, 43% versus 14% (p = 0.007); JAK1,2 inhibition attenuated the increase in SAA3 to 15% (p = 0.003). Urine albumin-to-creatinine ratio (r = 0.49, p = 0.03), mesangial index (r = 0.64, p = 0.001), and glomerulosclerosis score (r = 0.51, p = 0.02) were associated with SAA3 immunostaining scores across mouse groups. Exposing podocytes to AGE or exogenous SAA increased JAK2 activity within one hour and mRNA for associated inflammatory genes after 20 hours. JAK2 inhibition reduced SAA3 mRNA expression in podocytes exposed to AGE or SAA. SAA3 knockout podocytes had >85% lower AGE-induced inflammatory genes.

**Conclusion:**

JAK1,2 inhibition reduced SAA and histological features of DKD in podocyte JAK2-overexpressing mice. In podocytes exposed to a diabetes-like condition, JAK2 inhibition reduced expression of SAA, while SAA knockout blocked expression of associated pro-inflammatory mediators. SAA may promote JAK2-dependent inflammation in the diabetic kidney.

## Introduction

Pro-inflammatory mediators in the diabetic kidney are induced by activation of various signaling cascades [1–3]. Comparison of transcriptional networks between humans with diabetes and corresponding mouse models identified shared signals in the diabetic kidney [4, 5]. These signals, and their downstream pro-inflammatory mediators, provide candidate targets for new therapeutics for diabetic kidney disease (DKD). Janus kinase 2 (JAK2) is an intracellular tyrosine kinase that transduces cytokine-mediated signals. Compared to non-diabetic individuals, JAK2 is expressed at higher levels in the glomeruli and tubulointerstitium of patients with DKD [5]. In diabetic mice, podocyte-specific JAK2 overexpression exacerbated histological features of DKD and produced a phenotype similar to human DKD [6].

Serum amyloid A (SAA) is a family of acute phase reactants that exert numerous pro-inflammatory actions in many tissues and cells including the kidney [7, 8]. SAA is expressed in mice as acute-phase isoforms 1,2 and 3. Isoforms 1 and 2 are largely homologous (SAA1,2) while isoform 3 (SAA3) is more distinct and highly expressed in the kidney [7, 8]. In non-kidney tissue, SAA expression is up-regulated by JAK signaling. In synovial fibroblasts from the joints of patients with rheumatoid arthritis, JAK2 inhibition abrogated IL-6 induced expression of SAA mRNA [9]. Additionally, a non-specific JAK inhibitor reduced blood levels of SAA and associate pro-inflammatory mediators among patients with rheumatoid arthritis [10, 11]. In humans with advanced diabetic kidney disease, 24 weeks of JAK1/2 inhibition with baricitinib reduced albuminuria by 41% while also reducing circulating levels of SAA and other inflammatory biomarkers.[12]. Inflammation is clearly involved in DKD progression, and emerging evidence suggests that increased SAA production in the kidney promotes damage characteristic of DKD [7, 8]. SAA is up-regulated in cells by exposure to diabetes-like conditions (e.g. advanced glycation end products: AGE). Moreover, SAA itself induces pro-inflammatory responses in glomerular podocytes and mesangial cells [7]. Amounts of SAA mRNA are increased in both glomerular and tubulointerstitial compartments in kidneys from patients with DKD compared to non-diabetic glomerular disease and normal controls [7]. SAA protein also associated with histological severity of DKD in patients with both types 1 and 2 diabetes [7]. Similar findings have been observed in two experimental models of DKD, C57BL/6 streptozotocin mice (type 1 diabetic model) and BTBR *ob/ob* mice (type 2 diabetic model) [7].

Relationships between SAA and JAK2 signaling in the pathophysiology of DKD have not been previously explored. Currently there is a gap in the knowledge of how JAK2 inhibition attenuates inflammation in the diabetic kidney and which downstream effectors are involved. The study aims were to elucidate: 1) a JAK2-mediated pathway for increased SAA in the kidneys of diabetic mice; 2) a JAK2-SAA pathway for inflammation in podocytes.

## Methods

### Mouse model of diabetes

Podocyte JAK2-overexpressing 129S6 Akita mice (JAK2 mice; S1 Fig) were generated from a previous study and stored samples and data were used for the present study [6]. Non-diabetic wild-type (WT; n = 8) and diabetic WT (n = 8) controls, podocyte JAK2-overexpressing non-diabetic (n = 8) and diabetic (n = 15) male mice were implanted with Alzet osmotic minipumps (Model 1004; ALZA Scientific Products, Mountain View, California, USA) at 10 weeks of age [6]. Sterile 0.9% sodium chloride solution containing angiotensin II was infused at a rate of 700 ng/min/kg to accelerate pathologic changes in the kidney and produce a sufficiently severe phenotype for DKD in which to test JAK2 inhibition. Podocyte JAK2-overexpressing non-diabetic mice (n = 4) and diabetic mice (n = 9) also received a JAK1,2 inhibitor (compound LY03103801; Eli Lilly and Company, www.lilly.com) orally at a dose of 3 mg/kg/day in supplemental water (citric acid, pH 3.2) for the last two weeks of the angiotensin II infusion period (S1 Fig). Other mice received only supplemental water at the same volume. Mouse care and procedures were approved by the University of Michigan Committee on the Use and Care of Animals. Veterinary care was provided by the University of Michigan Unit for Laboratory Animal Medicine.

### Analytes in mouse urine and serum

Urine and blood samples were collected prior to euthanasia. Blood levels of glycated hemoglobin, triglycerides, and cholesterol were measured by the Michigan Diabetes Research Center Chemistry Laboratory [6]. Serum and urine samples were stored at −70°C for other assays [6]. Mouse SAA3 was measured by ELISA in serum (Mouse Serum Amyloid A-3 kit, EMD Millipore, www.millipore.com). Urinary albumin concentration was determined by ELISA (Cat. No. 1011, Albuwell, www.exocell.com), and urinary creatinine measurements were performed by a picric acid based assay (www.tecodiagnostics.com).

### Histological studies of mouse kidneys

Under general anesthesia, both kidneys were flushed under constant 100 mm Hg pressure with phosphate buffered saline (PBS) containing sodium heparin (50 U/ml) through a cannula placed in the abdominal aorta. The left kidney was ligated while the right kidney was perfused with a ferric oxide slurry for later isolation of glomeruli. The left kidney was removed and weighed. Kidney cortical regions were dissected and snap frozen in liquid nitrogen, or fixed overnight in a solution of 2% paraformaldehyde in PBS. Paraffin-embedded, paraformaldehyde-fixed tissue sections were stained with PAS (periodic acid-Schiff, immunostained with WT-1 antibodies to identify podocytes, and counter-stained with picrosirius red. Fifteen glomeruli per mouse were chosen randomly for quantification of glomerulosclerosis by proportionally scoring areas positive for PAS staining (MetaMorph Imaging Software version 6.1; Molecular Devices Corporation, Downingtown, PA, www.moleculardevices.com) and mesangial index [6].

### SAA protein in mouse kidneys

Immunostaining for mouse SAA3 and SAA1,2 proteins were performed with SAA isoform-specific rabbit antibodies [7, 13, 14]. Non-immune rabbit IgG (Sigma Chemical Co, www.sigmaaldrich.com) was used as a negative control. Tissue sections from paraffin-embedded, paraformaldehyde-fixed kidneys were de-paraffinized, hydrated, and subjected to antigen retrieval for 15 minutes at 97°C in citrate buffer, pH 6.0 (Vector laboratories, Inc., www.vectorlabs.com). Kidney sections were incubated overnight at 4° with anti-SAA3 (1:500) or anti-SAA1,2 (1:400) and non-immune rabbit IgG antibodies (5 ug/ml). Bound primary antibodies were detected by incubation with Impress-HRP Reagent (Vector laboratories, Inc.) for one hour at room temperature followed by 3,3’-diaminobenzidine. Kidney tissue sections were counterstained with hematoxylin.

SAA3 Immunostaining was assessed by two masked observers on 4 to 7 mice per group (two kidney sections per mouse) and 5 randomly-selected areas per section for scoring of abundance and intensity. Scoring for tubulointerstitial staining was based on staining area (0, 25, 50, 75, or 100% of visual field) and intensity (0—none; 1—light; 2—medium; and 3—darkest) [7]. The immunostaining score was the product of area and intensity. Scoring for glomerular staining was based on the proportion of positively stained glomeruli from 10 random glomeruli per section and two sections per mouse. Scores of two observers were averaged for a final score for tubulointerstitial and glomerular staining [7].

### RNA analyses in mouse glomeruli

RNA was harvested using the RNeasy Mini Kit with QIAshredder (Qiagen, www.qiagen.com) from glomeruli of podocyte JAK2-overexpressing diabetic mice (n = 12). Half of these mice (n = 6) had received the JAK1,2 inhibitor and the other half (n = 6) had received vehicle alone for two weeks. Gene expression profiling was performed by the Affymetrix Mouse Gene 2.1 ST platform. Image CEL files were log-transformed, analyzed at the single probe level, and summarized at the gene level with Chip Inspector [5].

### Mouse podocyte cell culture

Immortalized mouse podocytes (gift from Stuart Shankland, University of Washington) were grown on Collagen I (BD Biosciences, www.bdbiosciences.com) coated Primaria plates (VWR, www.vwrsp.com) in Roswell Park Memorial Institute (RPMI) 1640 medium (Sigma Chemical Company) [15]. Podocytes were grown at 33°C in media containing 10% heat inactivated fetal bovine serum (Atlas Biologicals Incorporated, www.atlasbio.com) and interferon-gamma (50 units/mL). Cell differentiation was promoted by incubation at 37°C in media without interferon-gamma for 8 to 10 days. Podocytes tested positive for nephrin by real-time PCR after differentiation. Podocytes were placed on RPMI with reduced fetal bovine serum (0.5%) for one day prior to exposure to exogenous SAA, AGE or control conditions (1 hour for JAK2 activity; 24 hours for mRNA assays). To make AGE-BSA, fatty acid-free fraction IV BSA (Sigma) was incubated with 0.5 M glucose for 45 days at 37°C. The resulting AGE-BSA solution was dialyzed with phosphate-buffered saline (PBS, pH 7.4) and sterile filtered; endotoxin was undetectable (E-TOXATE, Sigma). For exogenous SAA, recombinant human SAA1 protein (rSAA), (#300–53, PeproTech, www.peprotech.com) was used at 10 μg/mL [7]. AGE-bovine serum albumin was used at 300 ug/mL [7]. The JAK2 inhibitor, Tyrphostin AG 490 (50 μM; LC Laboratories, www.lclabs.com) was added to media along with experimental conditions.

### SAA3 knockout by CRIPSR/Cas9 in mouse podocytes

Knockout of SAA3 in podocytes was achieved by the CRISPR/Cas9 system. One 23-base sgRNA (5′-GAACTATGATGCTGCCCGGA-3′) was designed to the target site, exon three of the SAA3 gene (chromosome 7: NC_000073.6; Gene ID: 20210). The sgRNA expression cassette was driven by a U6 promoter. The T7 promoter was added upstream of the Cas9 sequence. mCherry, a fluorescent tag, was engineered into the vector controlled under an Sv40 promoter. All sequences were synthesized by Genecopeia (www.gencopeia.com). Podocytes were transfected according with a Lipofectamine 3000 (Invitrogen, www.thermofisher.com) protocol at 60% confluency in 6-well plates with 340 ng/μL of plasmid and 1.5 μL Lipofectamine 3000. After 48 hours of transfection, cells seeded on coverslips were examined for mCherry expression by confocal microscopy (Leica SPE Confocal Microscope, www.leica-microsystems.com) to assess uptake of plasmid. After confirmation of suitable transfection, cells were placed on neomyocin-containing (500 ng/μL) medium for 10 days. Neomycin-resistant colonies were passaged using serial dilutions into 60 mm dishes. After 72 hours, individual colonies were selected using cloning rings for culture in 60 mm dishes and non-neomyocin containing media. Gene knockout was verified by the absence of SAA3 mRNA (real-time PCR) and SAA3 protein in the media (ELISA) after exposure to AGE (S2 Fig).

### Western blot for JAK2 and phosphorylated-JAK2 in mouse podocytes

Cell lysates were prepared from podocytes grown in 60 mm dishes. Cells were washed and scraped from the plate with ice cold PBS. Podocytes were pelleted by centrifugation at 1000 x g for 10 minutes. The cell pellets were lysed in RIPA buffer (50 mM Tris pH 8.0, 150 mM sodium chloride, 0.5 mM ethylenediaminetetraacetic acid, 1 mM dithiothreitol, 1% NP-40, 0.5% sodium deoxycholate, 0.1% sodium dodecyl sulfate) containing HALT protease and phosphatase inhibitors (Pierce, www.thermofisher.com). Soluble cell lysates were denatured in reducing sodium dodecyl sulfate sample buffer at 95°C for 5 minutes. Samples were electrophoresed through denaturing sodium dodecyl sulfate-polyacrylamide gels (4 to 20% gradient, Bio-Rad) and proteins were transferred to nitrocellulose membranes. The membranes were blocked in Tris-hydrochloric acid buffered saline, pH 7.2, containing 0.05% Tween 20, and 5% non-fat dry milk, for one hour and then incubated with primary antibodies overnight at 4°C at 1:1000 dilutions (JAK2 #D2E12; phosphorylated-JAK2 #C80C3, Cell Signaling Technology, www.cellsignal.com). Bound antibodies were detected with horseradish peroxidase-conjugated anti-rabbit IgG (Cell Signaling Technology), followed by detection using SuperSignal West Chemiluminescent Substrate (Pierce). Digitized images were analyzed using a Chemidoc Touch Imaging system and Image Lab software version 5.2.1 (BioRad Laboratories, www.bio-rad.com).

### RNA analyses for SAA3 and inflammatory mediators in mouse podocytes

Total podocyte RNA was isolated and DNAse-I-treated by the RNAqueousmicro kit (Thermofisher, www.thermofisher.com). RNA was quantified using the Quant-iTRiboGreen RNA Reagent and kit (Thermofisher). cDNA was synthesized from equal amounts of RNA using Superscript III (Thermofisher). Expression of mRNA for SAA3, C-X-C motif chemokine ligand 5 (Cxcl5), C-C motif chemokine ligand 2 (Ccl2) and C-C motif chemokine ligand 5 (Ccl5) was measured by quantitative real-time PCR on an Applied Biosystems 7900HT Fast RT-PCR System using SA Biosciences SYBR Green reagent (Thermofisher) Gene amplification results were quantified with Sequence Detection System v2.4 software (Applied Biosystems) and normalized to mouse TATA-box binding protein.

### Statistical analysis

Data were assessed for normality using the Shapiro-Wilk test and verified using a Q-Q plot. Data for the urinary albumin-to-creatinine ratio (UACR) was log-transformed for analysis and back-transformed for data presentation. Analysis of variance was used to evaluate differences between groups for normally distributed data: mouse body weight, log UACR, and JAK2 and phosphorylated-JAK2 protein. Tukey’s honest significant difference test was used for multiple comparisons of positive F-tests. Pairwise Wilcoxon rank sum tests with Hommel adjusted p-values were used for non-normally distributed data: blood levels of glycated hemoglobin, total cholesterol, triglycerides; kidney tissue mesangial index and SAA immunostaining score; and mRNA expression data from podocyte experiments. The Significance Analysis of Microarrays method that utilizes a false discovery rate-controlling procedure to generate q values was utilized for the glomerular RNA data generated from microarrays [16]. The threshold for statistical significance for all analyses in this study were set at a significance level of p<0.05. Statistical analyses were conducted using R version 3.12 and Chip Inspector.

## Results

### Characteristics of diabetic and non-diabetic mice

**Fig 1:**
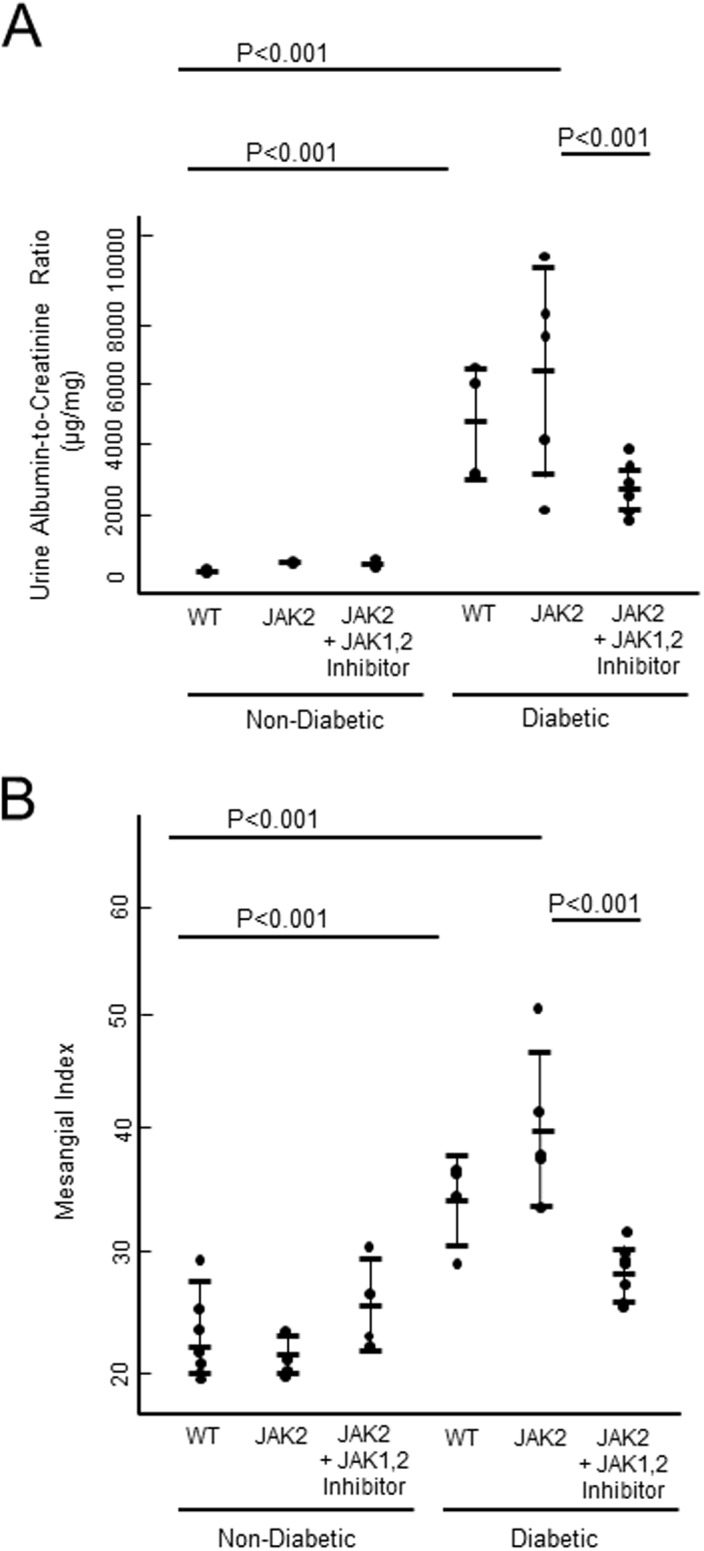
Albuminuria and structural markers of kidney damage in mice. A) UACR and B) mesangial index scores from non-diabetic and diabetic mice from wild type (WT) and podocyte specific JAK2 -overexpressing mice with and without the JAK1,2 inhibitor. WT non-diabetic control (n = 3), JAK2 non-diabetic control (n = 4), JAK2 non-diabetic + JAK1,2 inhibitor (n = 4), WT diabetic control (n = 4), JAK2 diabetic control (n = 6), and JAK2 diabetic + JAK1,2 inhibitor (n = 9).

Diabetic mice weighed less and had higher blood levels of glycated hemoglobin, total cholesterol, and triglycerides. JAK2 overexpression did not significantly affect these parameters (Table 1). Diabetes increased the UACR, but JAK2 overexpression did not significantly increase it. Treatment with the JAK1,2 inhibitor lowered UACR and the mesangial index in JAK2 mice (Fig 1).

**Table 1:** Body weight, glycemia, and lipids in mice.

	Non-Diabetic			Diabetic				
	Wild Type	JAK2	p-value^a^	Wild Type	p-value^a^	JAK2	p-value^a^	p-value^b^
**Body Weight (g)**	26 ± 2	26 ± 1	0.95	21 ± 2	<0.001	23 ± 2	0.025	0.27
**Glycated Hemoglobin (%)**	5 ± 0	5 ± 0	0.99	12 ± 2	<0.001	13 ± 2	<0.001	0.94
**Total Cholesterol (mg/dL)**	109 ± 5	112 ± 9	0.99	244 ± 108	<0.001	168 ± 50	0.27	0.10
**Triglycerides (mg/dL)**	90 ± 32	59 ± 16	0.42	137 ± 47	0.13	173 ± 66	0.004	0.38

^a^ versus non-diabetic wild-type

^b^ versus diabetic wild type

### SAA in the kidneys and blood of diabetic and non-diabetic mice

**Fig 2:**
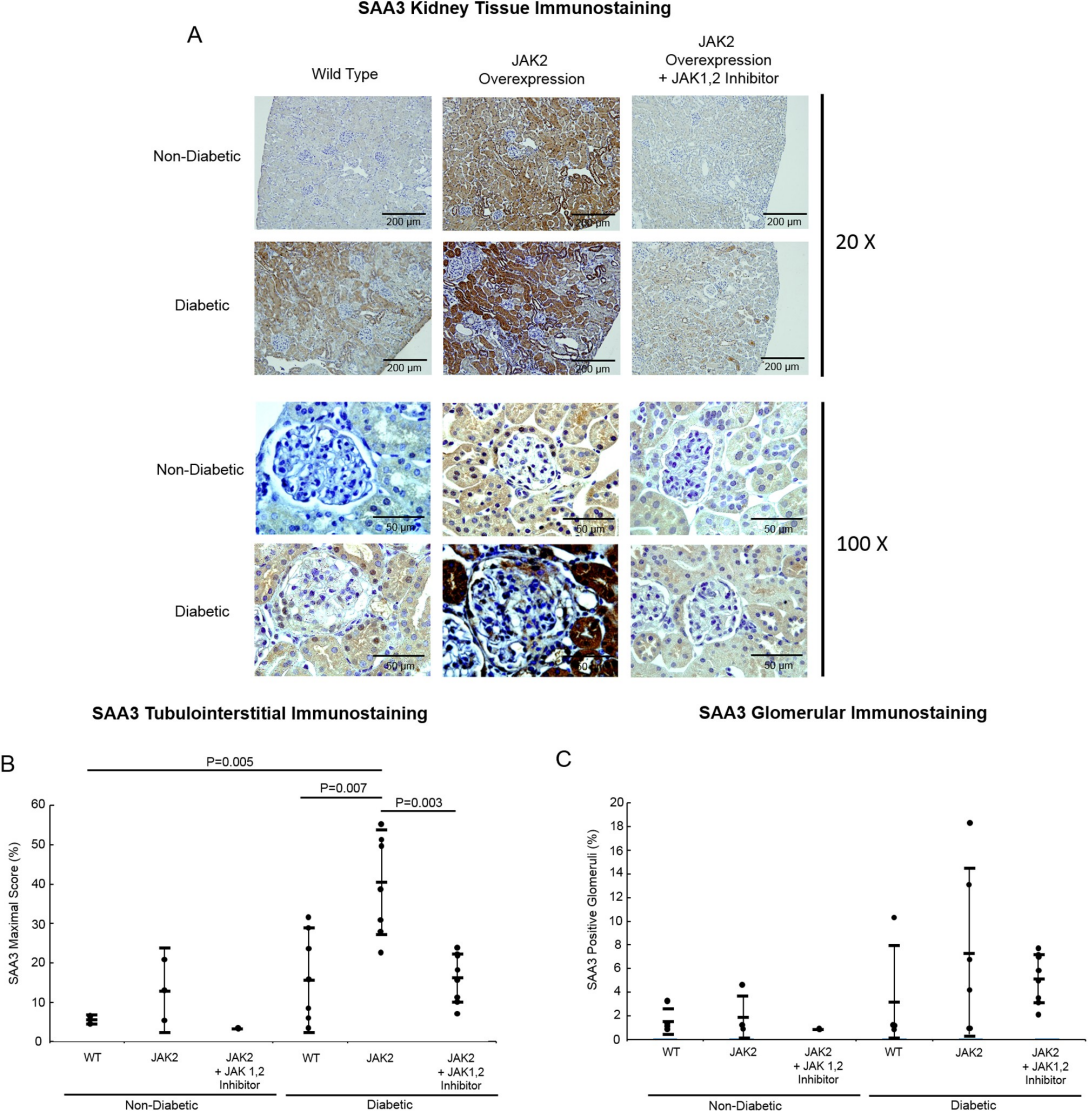
Mouse kidney immunostaining for SAA3 protein. Representative sections of non-diabetic and diabetic mouse kidneys from both wild type (WT) and podocyte specific JAK2-overexpressing mice with and without the JAK1,2 inhibitor. Magnification bars are shown in the lower right corner of each image. (B) SAA3 quantification by immunostaining scores in tubulointerstitium and (C) in glomeruli. WT non-diabetic control (n = 3), JAK2 non-diabetic control (n = 4), JAK2 non-diabetic with inhibitor (n = 4), WT diabetic control (n = 4), JAK2 diabetic control (n = 6), and JAK2 diabetic with JAK1,2 inhibitor (n = 9).

Kidney mRNA and protein for SAA3 along with serum SAA3 were analyzed to determine the effect of diabetes and JAK2 overexpression at the tissue and systemic levels, respectively. Diabetic JAK2 mice had quantifiably greater SAA3 protein in the tubulointerstitium compared to diabetic WT controls, 43% versus 14%. JAK1,2 inhibition attenuated the increases in SAA3 in JAK2 diabetic mice (15%, Fig 2). Differences in SAA3 protein in the glomeruli were not present by diabetes status or JAK2 overexpression (Fig 2). With JAK1,2 inhibition, SAA3 in the glomeruli of the JAK2 diabetic mice was similar to control WT diabetic and non-diabetic mice (Fig 2).

RNA was harvested from micro-dissected glomeruli of a subset of diabetic JAK2 mice that received either supplemental water (n = 6) or the JAK1,2 inhibitor (n = 6). In the diabetic JAK2 mice, JAK1,2 inhibition reduced SAA3 mRNA by 50% (q<0.01, n = 6 for each group) compared to the JAK2 diabetic mice that did not receive the inhibitor.

**Fig 3:**
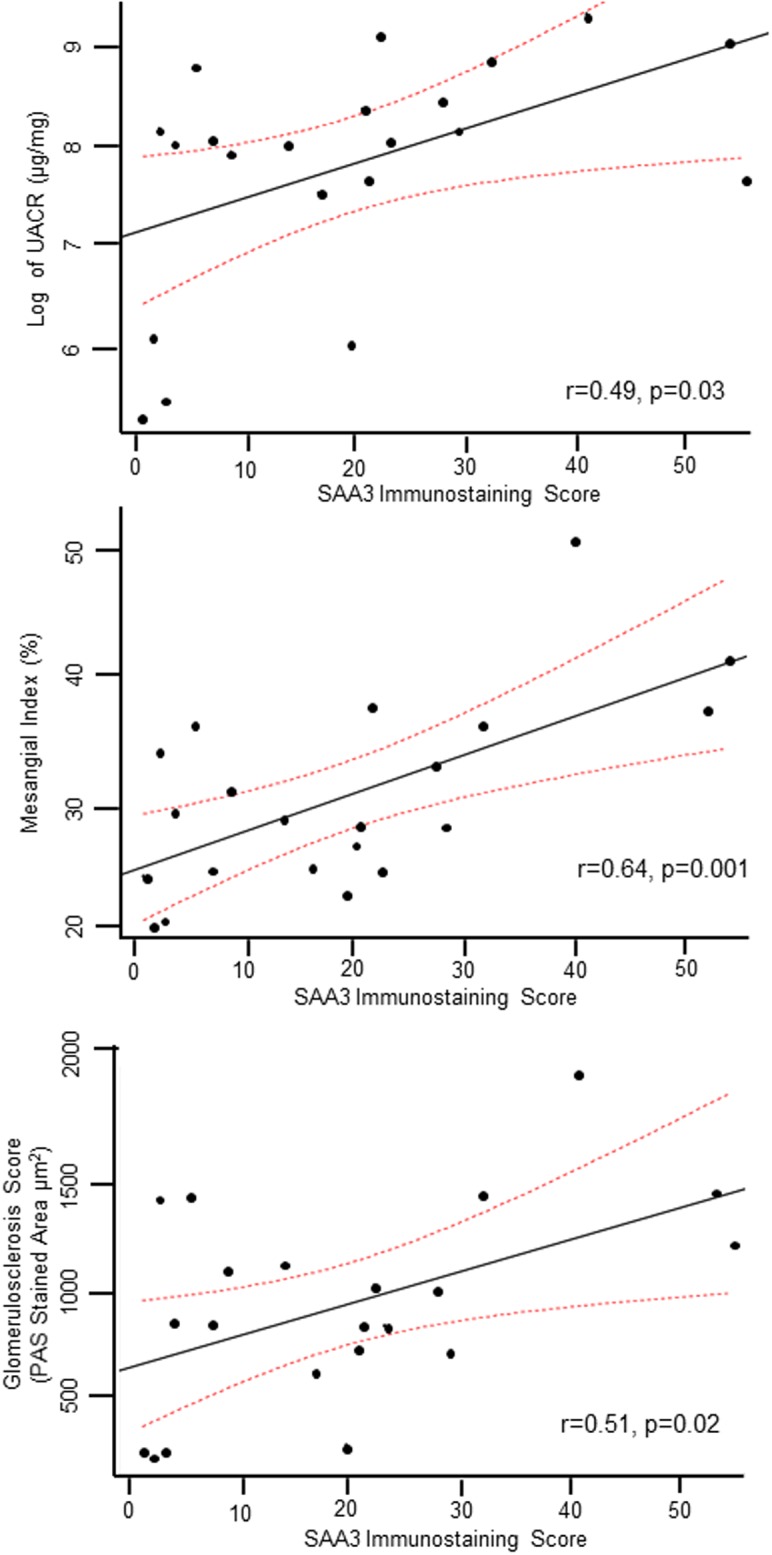
Associations of SAA protein with features of diabetic kidney disease. SAA protein was measured by the quantification of immunostaining and relationship were determined for to albuminuria (UACR), mesangial index (mesangial expansion), and glomerulosclerosis (PAS stained area) score. Mouse groups: WT Control (n = 3), JAK2 control (n = 4), JAK1,2 inhibitor (n = 4), WT diabetic control (n = 4), JAK2 diabetic control (n = 6) JAK2 diabetic with JAK1,2 inhibitor (n = 9).

**Fig 4:**
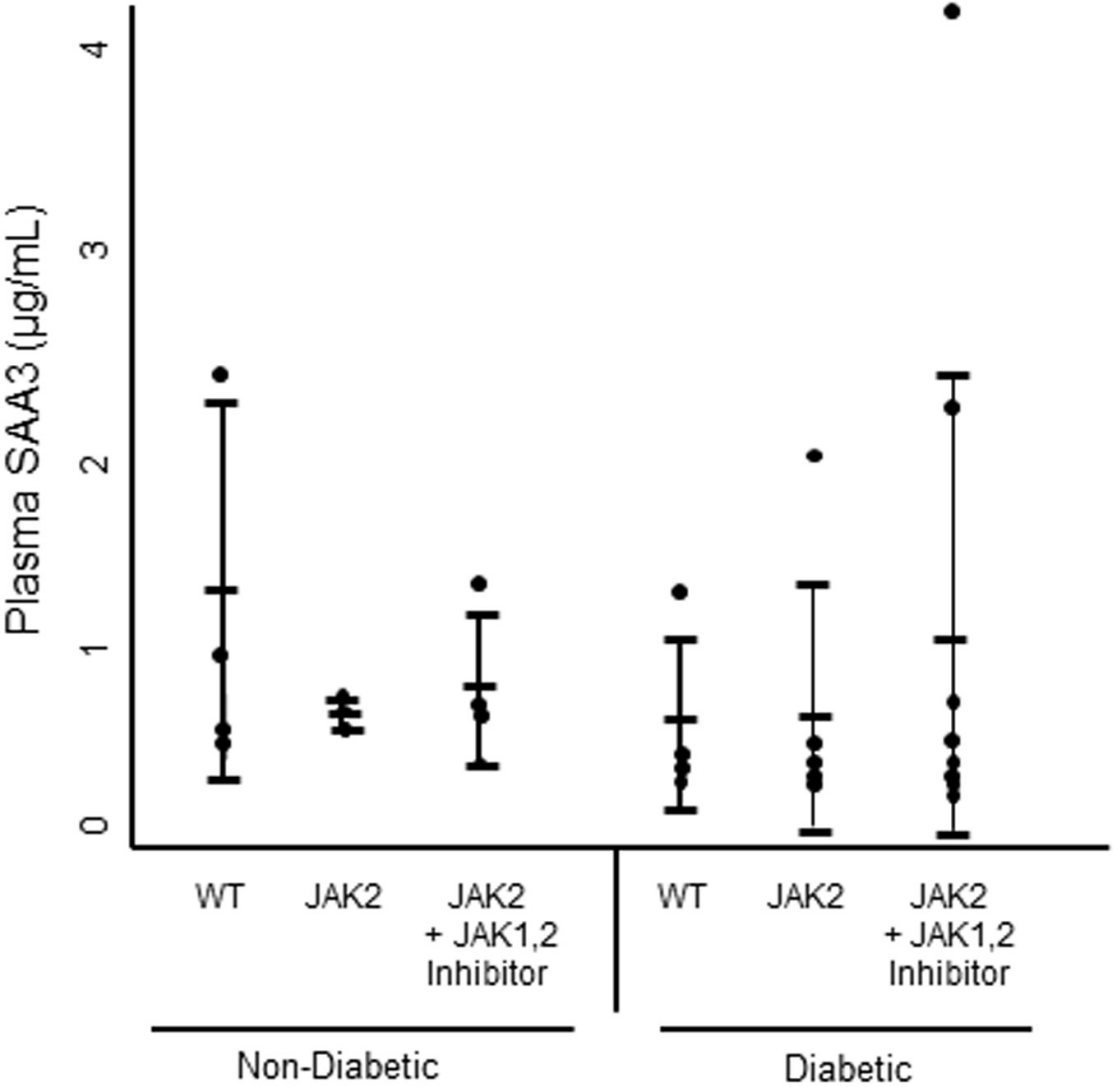
SAA3 protein concentrations in mouse plasma by diabetes and JAK2 status with or without the JAK1,2 inhibitor. Wild type (WT) control (n = 3), JAK2 control (n = 4), WT diabetic control (n = 4), WT diabetic + JAK1,2 inhibitor (n = 4), JAK2 diabetic control (n = 6) JAK2 diabetic + JAK1,2 inhibitor (n = 9).

Across mouse groups, SAA3 protein levels in the kidney correlated with indicators of glomerular damage: UACR, mesangial index, and glomerulosclerosis score, (Fig 3). Plasma SAA3 was not increased by diabetes or JAK2 overexpression (Fig 4).

### SAA and JAK2 signaling and inflammation in mouse podocytes

**Fig 5:**
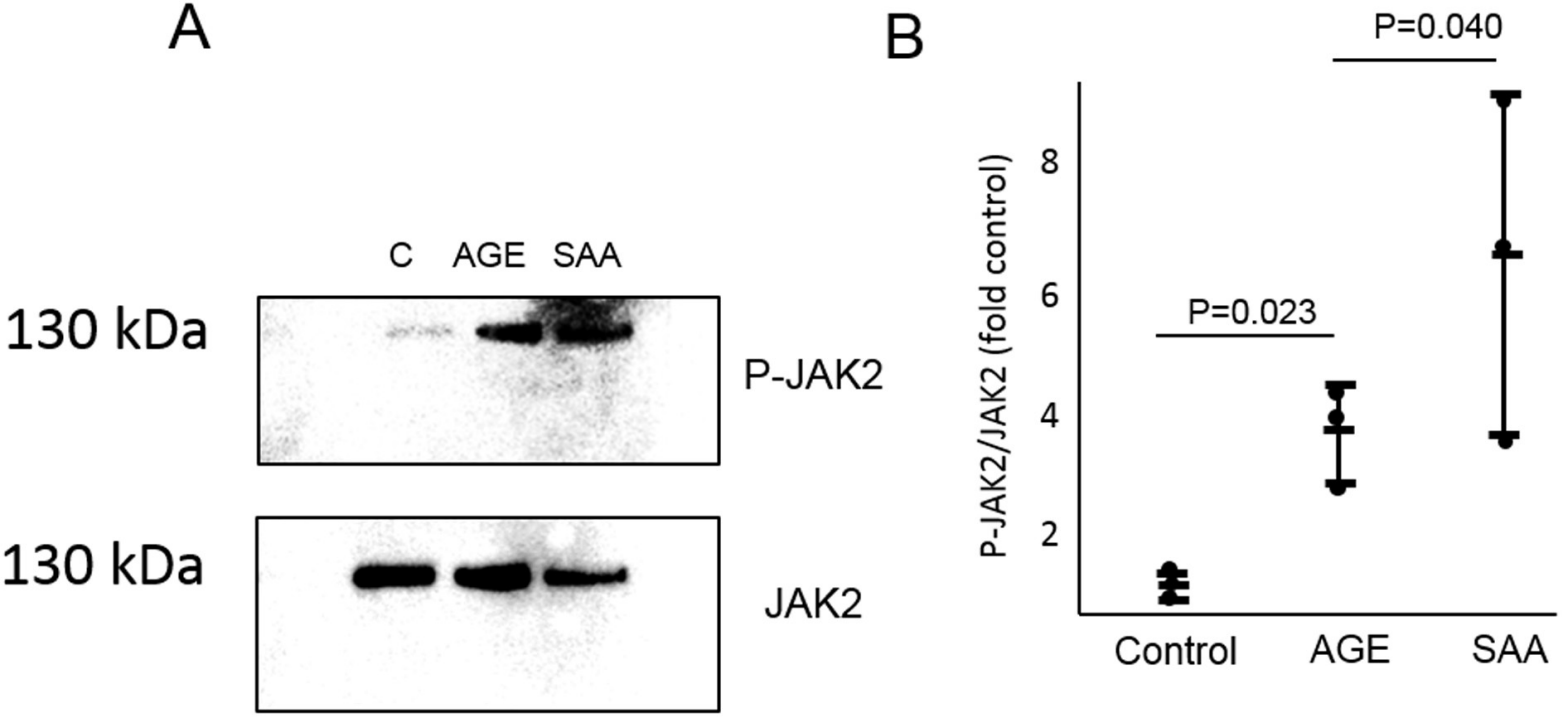
The effect of exposure to AGE and exogenous SAA on JAK2 activity. Podocytes were exposed to AGE or SAA for 1 hour. A) Representative Western blot showing phosphorylated and non-phosphorylated JAK2 (C: control sample). B) Quantitative data from Western blots showing effect of exposure to either AGE or SAA on JAK2 phosphorylation (n = 3 for each condition).

**Fig 6:**
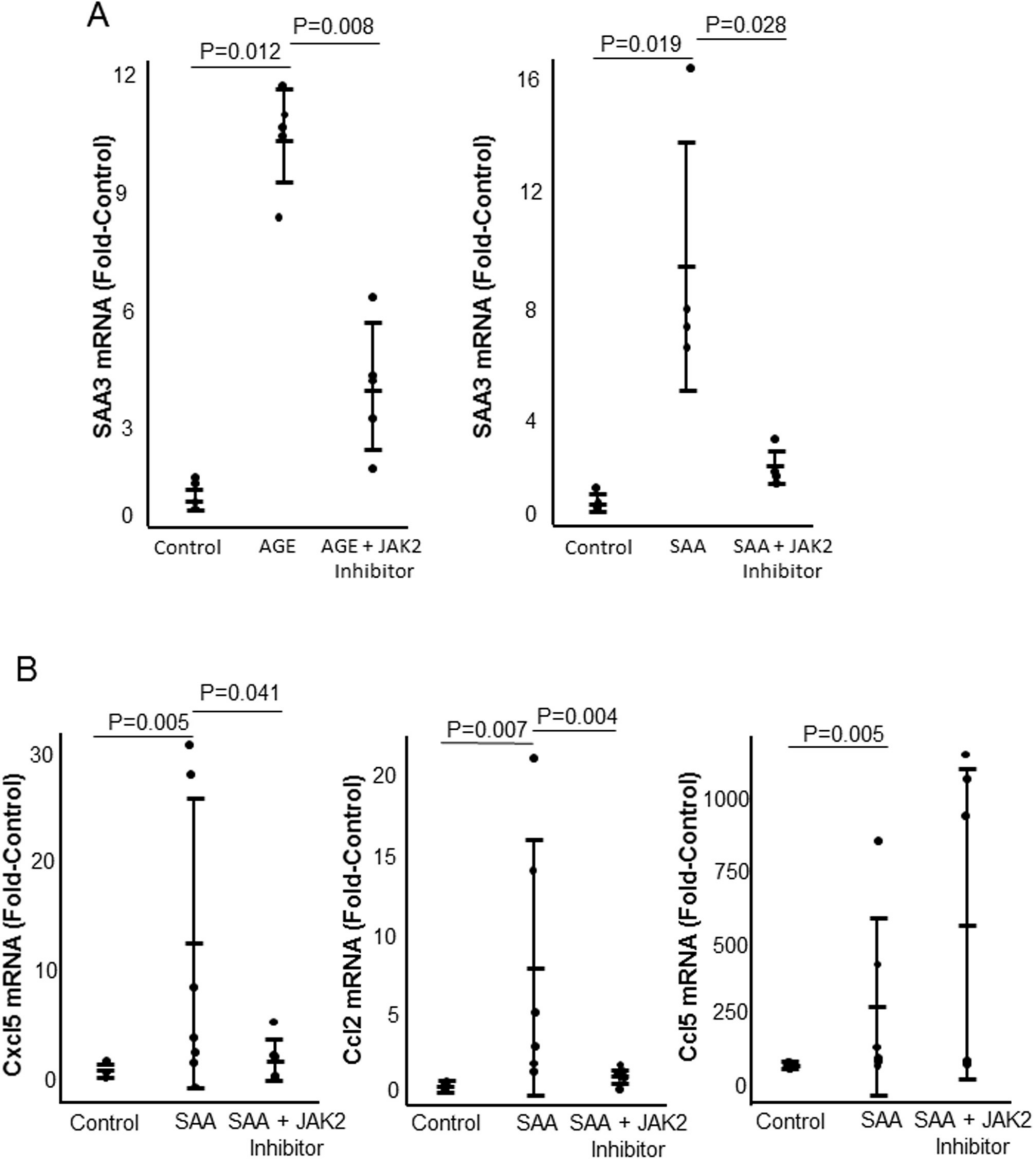
The effect of JAK2 inhibition on AGE or SAA induced inflammatory cytokine expression. Podocytes were exposed to AGE or SAA for 20 hours with or without the JAK2 inhibitor. A) Levels of SAA3 mRNA after exposure to AGE or SAA with and without the JAK2 inhibitor. B) Levels of SAA3, Cxcl5, Ccl2, and Ccl5 mRNA after exposure to SAA with or without the JAK2 inhibitor. n = 5 for each condition.

**Fig 7:**
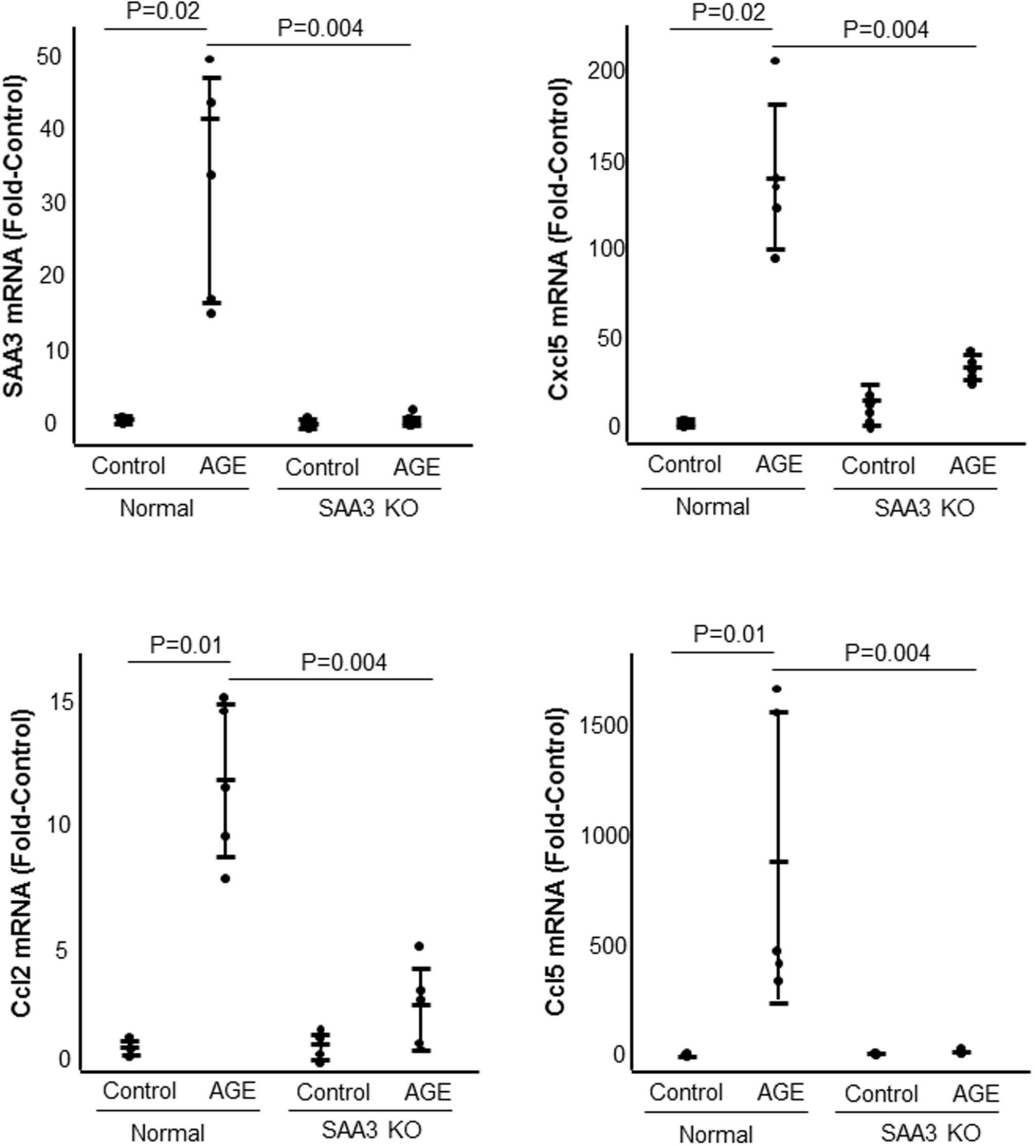
The effect of podocyte SAA3 knockout on AGE-induced SAA3 Cxcl5, Ccl2 and Ccl5 mRNA. Control and SAA3 knockout (KO) podocytes were exposed to AGE for 20 hours. n = 5 for each condition.

Exposure to AGE or SAA for 1 hour significantly increased JAK2 phosphorylation (Fig 5). Exposure to AGE or exogenous SAA significantly increased expression of SAA3 mRNA (Fig 6). Exposure of podocytes to exogenous SAA also significantly increased mRNA expression of Cxcl5, Ccl2, and Ccl5, while JAK2 inhibition significantly inhibited expression of Cxcl5 and Ccl2 but not Ccl5 (Fig 6). Control and SAA3 knockout podocytes were exposed to AGE for 20 hours. Knockout of SAA3 in podocytes significantly inhibited AGE-induced expression of SAA3, Cxcl5, Ccl2, and Ccl5 by greater than 85% (Fig 7).

## Discussion

Podocyte JAK2 overexpression in diabetes independently and synergistically increased SAA in the mouse kidney, which directly correlated with glomerular damage. Exposure to AGE or SAA activated JAK2 signaling to produce a pro-inflammatory response in podocytes, while knockout of SAA3 had a profound inhibitory effect on expression of JAK2-associated inflammatory mediators. Taken together, the present data show SAA to be a downstream mediator of JAK2 that may mechanistically contribute to podocyte-derived inflammation and consequent kidney damage in diabetes.

SAA3 protein was present in the tubulointerstitium and glomeruli of diabetic mice and increased by podocyte JAK2 overexpression. Non-diabetic mice did not have detectable SAA3 in the kidney except with podocyte JAK2 overexpression. In contrast, there was nominal immunostaining in the mouse kidneys for SAA1,2, the main systemic isoforms of SAA (S2 Fig). The present findings of SAA3 protein in the kidneys of this mouse model of type 1 diabetes expand our previous findings of robust amounts of SAA3 in the C57BL/6 streptozotocin type 1 diabetes model and the BTBR *ob/ob* type 2 diabetes model of DKD [7]. These data also correspond to the kidney-expressed human SAA isoform in patients with DKD due to either type 1 or type 2 diabetes [7]. JAK1,2 inhibition attenuated the amount of SAA3 protein in the tubulointerstitium of diabetic mice and JAK2 diabetic and non-diabetic mice. Furthermore, JAK1,2 inhibition reduced SAA3 mRNA expression by approximately 50% in glomeruli from diabetic JAK2 mice. In sum, locally-produced SAA3, rather than systemic SAA 1,2, is the primary isoform present in the diabetic mouse kidney [7].

Blood levels of SAA3 were not different between diabetic mice and their controls in the present study in contrast to previous observations [7]. This may be due to the infusion of angiotensin II, which induces expression of SAA3 in non-kidney tissues, and in this way, may increase systemic levels to mitigate between group differences [17]. However, despite similar levels of systemic SAA3 across experimental groups, marked increases in SAA3 protein in the kidneys of diabetic and/or podocyte JAK2-overexpressing mice are consistent with local production. These data support earlier findings that SAA3 is highly expressed in the mouse kidney and contributes minimally to circulating SAA [7, 14, 18]. Additionally, the present observations suggest that urine, a direct effluent from the kidney, may be a better sample source than blood to potentially target for SAA biomarker development [19, 20].

This study advances the understanding of how JAK2 is involved in the pathogenesis of DKD by identifying a particular downstream mediator, namely SAA and an associated inflammatory response. JAK2 is expressed at greater levels in the tubulointerstitium and glomeruli of humans with DKD compared to non-diabetic controls. Enhanced expression of JAK2 specifically in podocytes markedly augmented the DKD phenotype in the present model of type 1 diabetes in mice [5, 6]. In cultured podocytes, exogenous SAA induced JAK2 activity and SAA3 overexpression along with associated inflammatory mediators. Inhibition of JAK2 activity in podocytes reduced the AGE or exogenous SAA-induced expression of SAA3 and corresponding inflammatory mediators. Notably, SAA3 knockout in podocytes abolished AGE-induced expression of JAK2-upregulated inflammatory mediators. As such, increased SAA appears to be both a consequence and a cause of JAK2 signaling, and therefore, may drive a “feed forward” loop that leads to a broad inflammatory response.

Exposure of cultured podocytes to AGE, a diabetes-like condition, increased JAK2 activity and induced expression of SAA3 as well as pro-inflammatory mediators including Cxcl5, Ccl2, and Ccl5. In particular, Ccl2 is a central chemokine for tissue macrophage recruitment. A link between local SAA production and Ccl2 expression has been observed in other mouse models [21–23]. For example, in C57BL/6 mice fed a pro-inflammatory diet (high-fat, high-sucrose), knockout of SAA3 attenuated increases in Ccl2 expression and macrophage accumulation in visceral adipose tissue compared to control C57BL/6 mice fed the same diet [24]. Local production of SAA3 also induces Ccl2 and inflammation in adipose tissue of ob/ob mice [22]. Inhibition of JAK2 did not reduce SAA-induced CCL5, suggesting that there are JAK2 independent mechanisms by which inflammation is regulated. In sum, these data support the overall concept that local SAA expression is causal for tissue inflammation.

The present study integrates understanding of inflammation in DKD mediated by JAK signaling and SAA, thereby, identifying a novel mechanistic pathway. JAK2 is known to be up-regulated in the kidneys of humans with DKD. Independent work demonstrated both increased expression of SAA mRNA and protein associated with histological injury in the kidneys of humans with DKD [5,7]. In a randomized controlled clinical trial of patients with advanced DKD, administration of a JAK1/2 inhibitor reduced systemic levels of SAA with concurrent reductions in albuminuria. Thus, there is therapeutic evidence for JAK1/2 regulation of SAA in humans with DKD [12]. The present data provide further evidence that a JAK2-SAA pathway may be involved with direct effects on the kidney. Furthermore, JAK2-induced SAA may be responsible for driving a panoply of additional inflammatory mediators.

An inherent limitation of this study is that a mouse model does not fully replicate human DKD. However, podocyte JAK2 overexpression produced a more “humanized” representation of the diabetic kidney [6]. Moreover, the cross-species comparison of glomerular gene expression profiles between mice and humans provided a unique opportunity to determine the role of JAK2 and SAA in DKD. Specifically, the murine SAA3 gene is locally expressed in the kidney [25]. Mouse SAA3 is most comparable across sequence similarity, tissue expression, and biological function to the SAA isoforms found in the human diabetic kidney [26]. Therefore, the demonstration of local expression of SAA3 in the mouse diabetic kidney may allow translation of these findings into understanding the role of kidney-specific SAA isoforms in human DKD. Another limitation of the present study is that we did not observe significant increases in glomerular staining by JAK2 overexpression, the compartment in which JAK2 overexpression occur. However, we did see changes in the tubulointerstitial, which may indicate that our measurements were not sensitive enough to detect changes in immunostaining in the glomerular compartment. The use of AGEs in the *in vitro* studies is also a limitation as it does not fully recapitulate the *in vivo* environment, which also includes high glucose, high insulin, and myriad inflammatory mediators. However, the use of AGEs has shown to be a robust model of inducing inflammation in resident glomerular cells, specifically the expression of SAA [7,8].

In conclusion, SAA, a potent pro-inflammatory mediator, was uniquely present and increased in the kidneys of diabetic mice and further augmented by podocyte JAK2 overexpression. Inhibition of the JAK1,2 pathway ameliorated histological features of DKD in this mouse model. JAK2-dependent inflammatory mechanisms associated with diabetes were mediated by SAA in podocytes. Further investigation of JAK2-SAA associated inflammatory networks represent a promising area for therapeutic and biomarker development in DKD.

## Supporting information

S1 Table Primer sequences. (DOCX)

S1 Fig Depiction of target vector used to create podocyte JAK2 overexpressing mice and experimental protocol. (DOCX)

S2 Fig The effect of AGE exposure on SAA3 protein levels in wild-type and SAA knockdown cells. Wild type and SAA3 knockdown cells were exposed to AGE for 24 hours and media was harvested and analyzed for SAA3 protein content by ELISA. n = 3 for each condition.
(DOCX)

S3 Fig Mouse kidney immunostaining for SAA1,*2*. A) Representative sections of non-diabetic and diabetic mouse kidneys from wild type (WT) and JAK2 mice +/- the JAK1,2 inhibitor (LY03103801). B) Positive control for SAA1,2 antibody in kidneys from control and lipopolysaccharide (LPS) injected mice after 20 hours. Images taken at 20x.
(DOCX)

S4 Fig Expression of WT-1 mRNA in podocytes. Podocytes were differentiated and kept in culture for 7 days post differentiation and WT-1 mRNA levels were measured using RT-qPCR.
(DOCX)

S5 Fig Expression of SAA3 mRNA in microdissected glomeruli. RNA was harvested from glomeruli of podocyte JAK2-overexpressing diabetic mice who received vehicle (n = 6) or the JAK1,2 inhibitor (n = 6). Gene expression profiling was performed by the Affymetrix Mouse Gene 2.1 ST platform.
(DOCX)

S1 File Manuscript data. (ZIP)
