# Supplementary material for: Serum amyloid A and Janus kinase 2 in a mouse model of diabetic kidney disease
Source: PLoS One. 2019 Feb 14;14(2):e0211555. doi: 10.1371/journal.pone.0211555 (PMC6375550; doi:10.1371/journal.pone.0211555)
Supplement: S1 Table — (DOCX) [file pone.0211555.s001.docx]

**S1 Table**. **Primer sequences**

| **Mouse Gene Primer Sequences** | | |
| --- | --- | --- |
| **SAA3** | Forward | TGATGCTGCCCGGAGGGGTC |
|  | Reverse | CTCCGGCCCCACTCATTGGC |
| **Ccl2** | Forward | CCTGTCATGCTTCTGGGCCTGC |
|  | Reverse | GGGGCGTTAACTGCATCTGGCTG |
| **Ccl5** | Forward | ATATGGCTCGGACACCACTC |
|  | Reverse | TCCTTCGAGTGACAAACACG |
| **Cxcl5** | Forward | GAGGACTCTGACCCCAGTGA |
|  | Reverse | GGGACAATGGTTTCCCTTTT |
| **TATA-box binding protein** | Forward | TGCTGTTGGTGATTGTTGGT |
|  | Reverse | CTGGCTTGTGTGGGAAAGAT |
