# Supplementary material for: Serum amyloid A and Janus kinase 2 in a mouse model of diabetic kidney disease
Source: PLoS One. 2019 Feb 14;14(2):e0211555. doi: 10.1371/journal.pone.0211555 (PMC6375550; doi:10.1371/journal.pone.0211555)
Supplement: S1 Fig — (DOCX) [file pone.0211555.s002.docx]

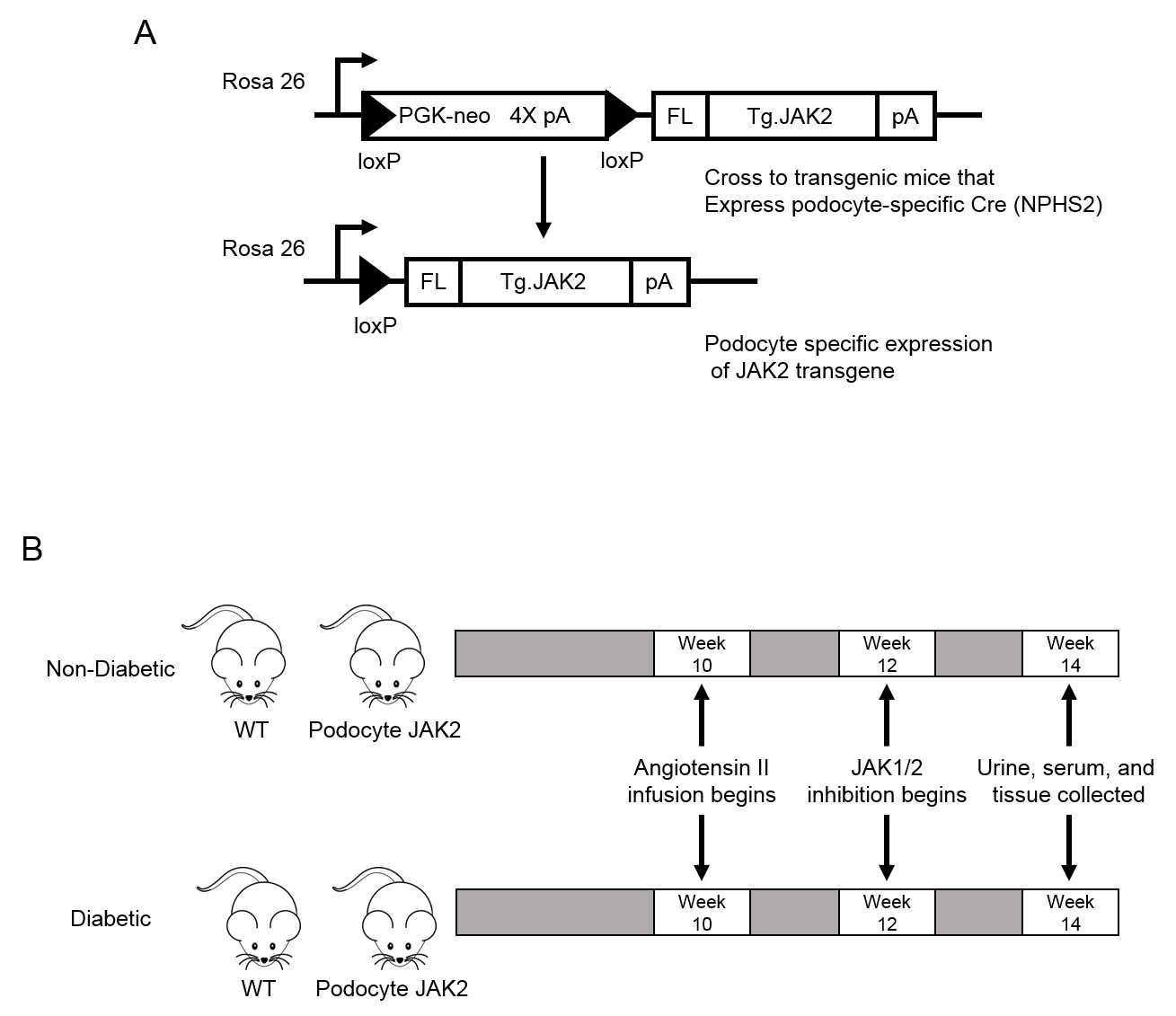
**S1 Fig.** **Depiction of target vector used to create podocyte JAK2 overexpressing mice and experimental protocol.**
