# Supplementary material for: Serum amyloid A and Janus kinase 2 in a mouse model of diabetic kidney disease
Source: PLoS One. 2019 Feb 14;14(2):e0211555. doi: 10.1371/journal.pone.0211555 (PMC6375550; doi:10.1371/journal.pone.0211555)
Supplement: S2 Fig — Wild type and SAA3 knockdown cells were exposed to AGE for 24 hours and media was harvested and analyzed for SAA3 protein content by ELISA. n = 3 for each condition. (DOCX) [file pone.0211555.s003.docx]

**S2 Fig. The effect of AGE exposure on SAA3 protein levels in wild-type and SAA knockdown cells.** Wild type and SAA3 knockdown cells were exposed to AGE for 24 hours and media was harvested and analyzed for SAA3 protein content by ELISA. n=3 for each condition
