# Supplementary material for: Serum amyloid A and Janus kinase 2 in a mouse model of diabetic kidney disease
Source: PLoS One. 2019 Feb 14;14(2):e0211555. doi: 10.1371/journal.pone.0211555 (PMC6375550; doi:10.1371/journal.pone.0211555)
Supplement: S3 Fig — A) Representative sections of non-diabetic and diabetic mouse kidneys from wild type (WT) and JAK2 mice +/- the JAK1,2 inhibitor (LY03103801). B) Positive control for SAA1,2 antibody in kidneys from control and lipopolysaccharide (LPS) injected mice after 20 hours. Images taken at 20x. (DOCX) [file pone.0211555.s004.docx]

**
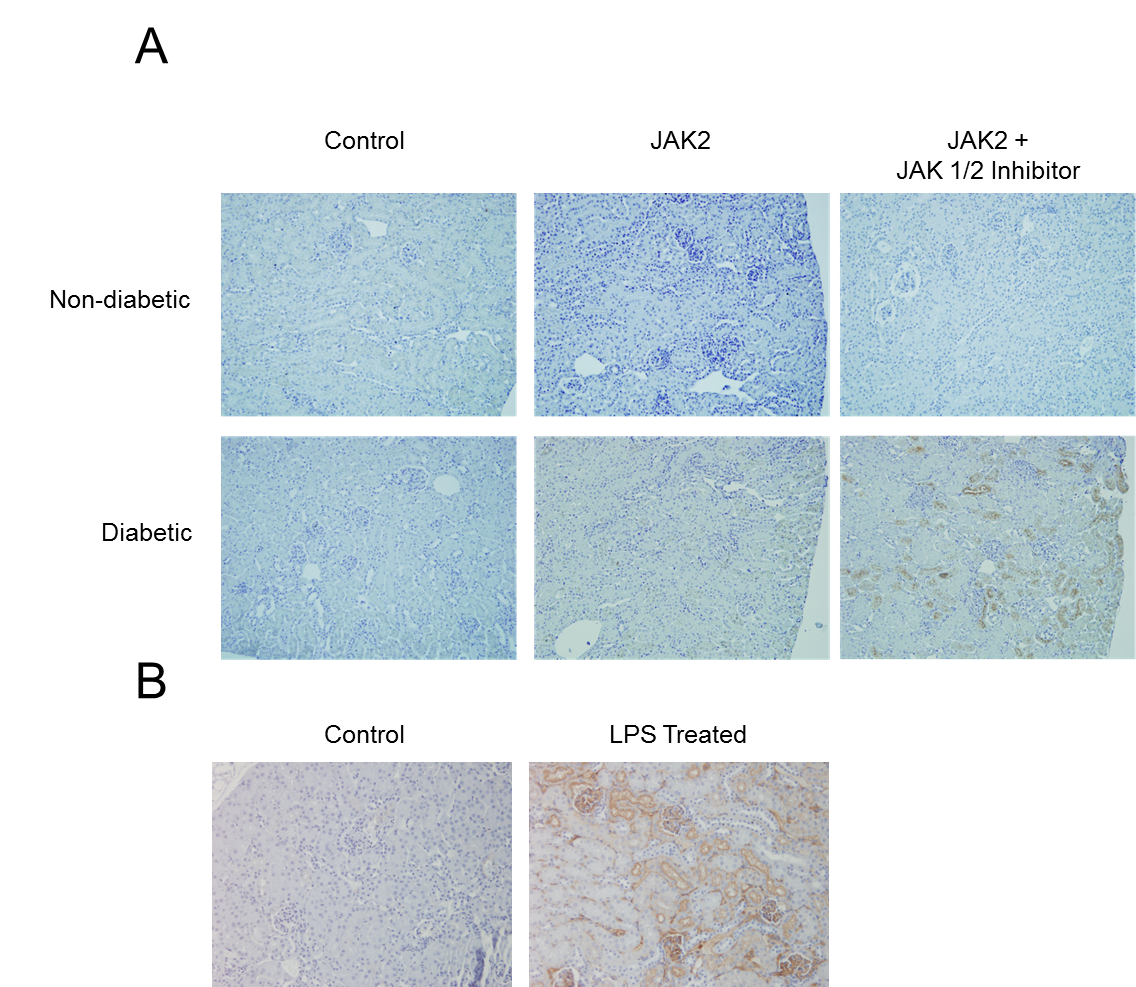
**

**S3 Fig. Mouse kidney immunostaining for SAA1*,2***. A) Representative sections of non-diabetic and diabetic mouse kidneys from wild type (WT) and JAK2 mice +/- the JAK1,2 inhibitor (LY03103801). B) Positive control for SAA1,2 antibody in kidneys from control and lipopolysaccharide (LPS) injected mice after 20 hours. Images taken at 20x.
