# Supplementary material for: Serum amyloid A and Janus kinase 2 in a mouse model of diabetic kidney disease
Source: PLoS One. 2019 Feb 14;14(2):e0211555. doi: 10.1371/journal.pone.0211555 (PMC6375550; doi:10.1371/journal.pone.0211555)
Supplement: S4 Fig — Podocytes were differentiated and kept in culture for 7 days post differentiation and WT-1 mRNA levels were measured using RT-qPCR. (DOCX) [file pone.0211555.s005.docx]

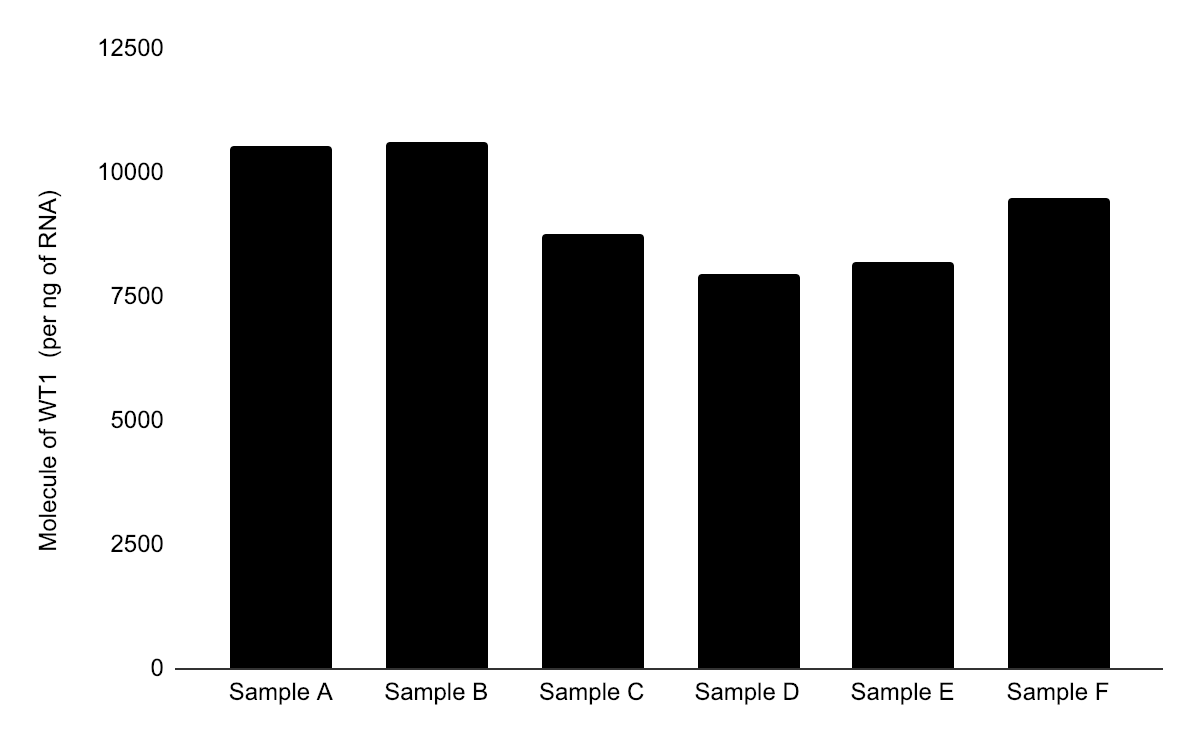


**S4 Fig. Expression of WT-1 mRNA in podocytes**. Podocytes were differentiated and kept in culture for 7 days post differentiation and WT-1 mRNA levels were measured using RT-qPCR.
