# Supplementary material for: Serum amyloid A and Janus kinase 2 in a mouse model of diabetic kidney disease
Source: PLoS One. 2019 Feb 14;14(2):e0211555. doi: 10.1371/journal.pone.0211555 (PMC6375550; doi:10.1371/journal.pone.0211555)
Supplement: S5 Fig — RNA was harvested from glomeruli of podocyte JAK2-overexpressing diabetic mice who received vehicle (n = 6) or the JAK1,2 inhibitor (n = 6). Gene expression profiling was performed by the Affymetrix Mouse Gene 2.1 ST platform. (DOCX) [file pone.0211555.s006.docx]

**S5 Fig. Expression of SAA3 mRNA in microdissected glomeruli.** RNA was harvested from glomeruli of podocyte JAK2-overexpressing diab
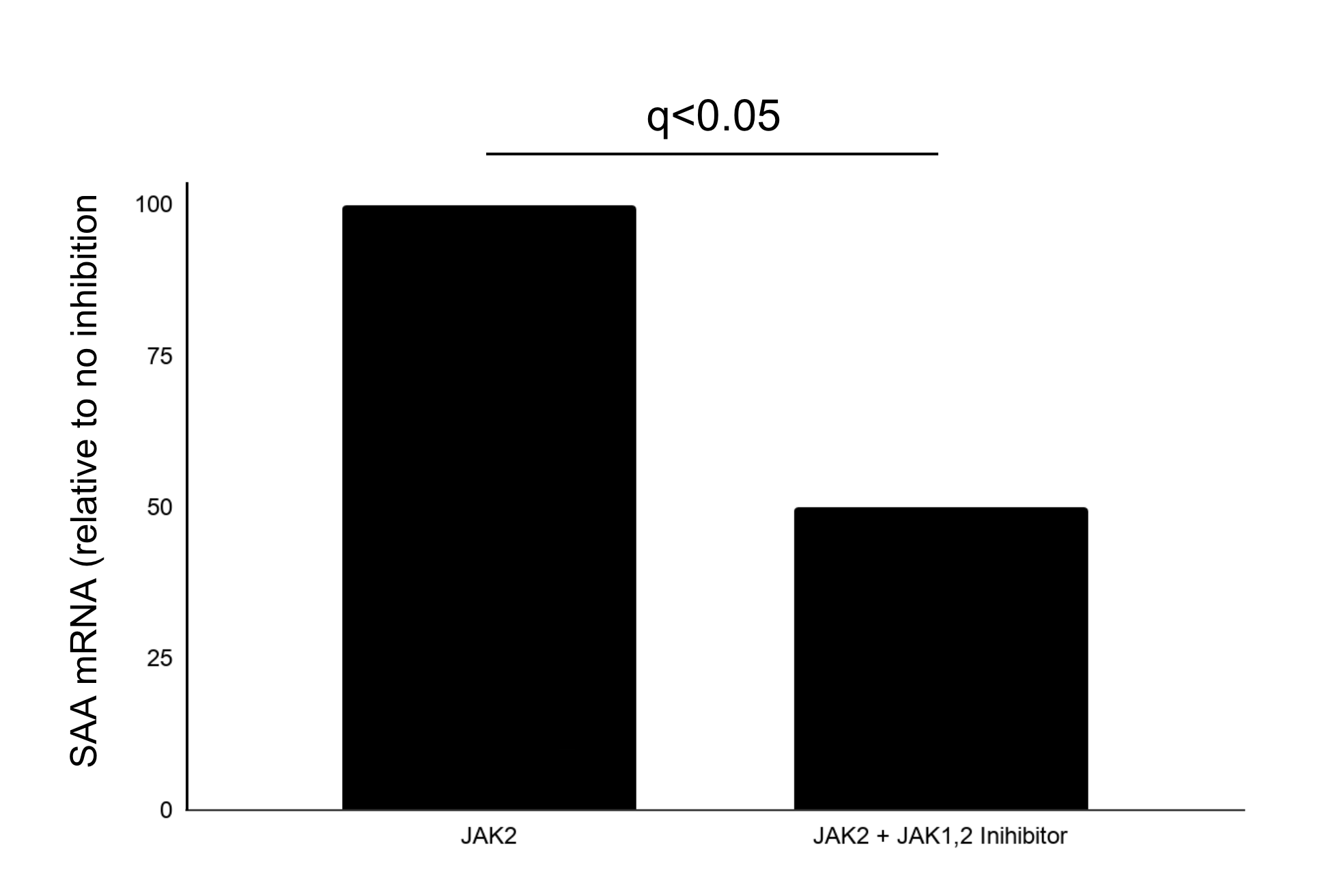
etic mice who received vehicle (n=6) or the JAK1,2 inhibitor (n=6). Gene expression profiling was performed by the Affymetrix Mouse Gene 2.1 ST platform.
